# Engineering gold-platinum core-shell nanoparticles by self-limitation in solution

**DOI:** 10.1038/s42004-022-00680-w

**Published:** 2022-06-06

**Authors:** Marc Ledendecker, Paul Paciok, Wojciech T. Osowiecki, Marc Pander, Marc Heggen, Daniel Göhl, Gaurav A. Kamat, Andreas Erbe, Karl J. J. Mayrhofer, A. Paul Alivisatos

**Affiliations:** 1 Department of Technical Chemistry I, Technical University Darmstadt, Alarich-Weiss-Straße 8, 64287 Darmstadt, Germany; 2 Ernst Ruska-Centre for Microscopy and Spectroscopy with Electrons and Peter Grünberg Institute, Forschungszentrum Jülich GmbH, 52425 Jülich, Germany; 3 Department of Chemistry, University of California, Berkeley, California, CA 94720 USA; 4 Department of Interface Chemistry and Surface Engineering, Max-Planck-Institut für Eisenforschung GmbH, Max-Planck-Straße 1, 40237 Düsseldorf, Germany; 5 Department of Materials Science and Engineering, NTNU, Norwegian University of Science and Technology, 7491 Trondheim, Norway; 6 Forschungszentrum Jülich, Helmholtz-Institute Erlangen-Nürnberg for Renewable Energy (IEK-11), Egerlandstraße 3, 91058 Erlangen, Germany; 7 Department of Chemical and Biological Engineering, Friedrich-Alexander-Universität Erlangen-Nürnberg, Egerlandstraße 3, 91058 Erlangen, Germany; 8 Department of Materials Science and Engineering, University of California, Berkeley, CA 94720 USA; 9 Kavli Energy NanoScience Institute, Berkeley, CA 94720 USA

**Keywords:** Electrocatalysis, Nanoparticles, Energy science and technology, Synthesis and processing, Synthesis and processing

## Abstract

Core-shell particles with thin noble metal shells represent an attractive material class with potential for various applications ranging from catalysis to biomedical and pharmaceutical applications to optical crystals. The synthesis of well-defined core-shell architectures remains, however, highly challenging. Here, we demonstrate that atomically-thin and homogeneous platinum shells can be grown via a colloidal synthesis method on a variety of gold nanostructures ranging from spherical nanoparticles to nanorods and nanocubes. The synthesis is based on the exchange of low binding citrate ligands on gold, the reduction of platinum and the subsequent kinetically hindered growth by carbon monoxide as strong binding ligand. The prerequisites for homogeneous growth are low core-binding ligands with moderate fast ligand exchange in solution, a mild reducing agent to mitigate homonucleation and a strong affinity of a second ligand system that can bind to the shell’s surface. The simplicity of the described synthetic route can potentially be adapted to various other material libraries to obtain atomically smooth core-shell systems.

## Abstract (web summary)

Core-shell particles with thin noble metal shells represent an attractive material class with potential for various applications ranging from catalysis to biomedical applications, but the synthesis of well-defined core-shell architectures remains highly challenging. Here, the authors report the chemically induced self-limiting growth of atomically-thin and homogeneous platinum shells on a variety of gold nanostructures.

## Introduction

Core-shell nanostructures can be used as selective and active catalysts, for biomedical and pharmaceutical applications, and for the creation of photonic crystals and high photoluminescent materials^1–8^. A number of strategic routes to obtain these structures are known in literature, such as galvanic displacement^9–12^, laser ablation^13^, plasma sputtering^14^ controlled reduction^15–18^, exploitation of thermodynamic segregation through annealing^19,20^, anion coordination^21^ or through a SiO_2_ encapsulated nanoreactor approach^7^. The synthesis, however, demands often the use of organic solvents, multiple synthesis steps, direct electrode contact, high temperatures, or multiple nanometers in shell thickness to obtain a continuous layer^22,23^. As the metal ion diffusion to the surface is often the rate limiting step, galvanic displacement can result in alloy formation or in a porous structure^24,25^. Additionally, homogeneous shell growth is challenging as nucleation and growth preferentially occur on vertices or edge sites with low coordination numbers and high surface energies^26^. In literature, various bimetallic nanostructures have been synthesized^27–29^. The overgrowth, however, resulted often in separated nanoparticles that nucleated first at e.g. the endcaps and edges of Au-rods. Moffat and coworkers reported an electrochemically induced self-terminating growth on film substrates^30,31^. Here, platinum was electrodeposited on bare gold electrodes in the presence of a strongly binding surfactant. Similarly, by atomic layer deposition, Jaramillo, Prince and co-workers altered the specific binding energies between metal and functional groups and the growth of 2-dimensional (2D) layers on polycrystalline films has been described^32^. The main challenges, however, are full Pt-coverages on the underlying substrate and a general applicability to tailored structures and nanoparticle dimensions. On the nanoscale, surfactants bind to the nanoparticle surface, provide colloidal stability and can alter the material’s surface energy and polarity. Potentially, the use of complementary surfactant systems allows for controlling the kinetic and growth characteristics of core-shell structures with tailored properties. Here, we introduce a synthesis concept that relies on two complementary metal-ligand systems resulting in various core-shell nanostructures ranging from spherical nanoparticles to nanorods and nanocubes. Note that within this work, the nanoparticle interior is termed “core”, while the term “shell” refers to the outer metal layer independently of the nanoparticle aspect ratio.

## Results and discussion

**Fig. 1:**
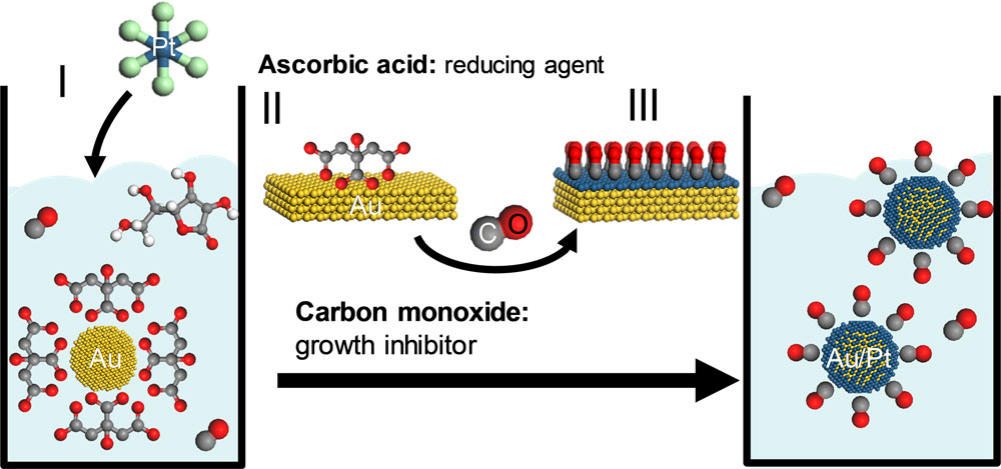
Conceptual pathway of the chemically induced self-limiting growth of Pt on Au nanoparticles. To a CO-saturated solution of citrate capped gold nanoparticles and ascorbic acid, H_2_PtCl_6_ is added (I). Ascorbic acid is oxidized on the Au-surface while Pt is reduced (II). After Pt-deposition, CO acts as growth inhibitor resulting in core-shell nanoparticles (III).

**Fig. 2:**
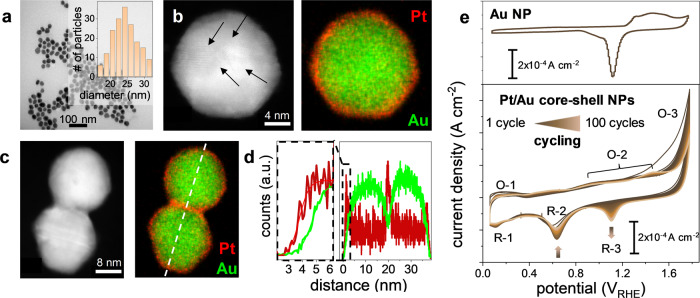
Characterisation of Pt/Au core-shell nanoparticles. TEM images of Pt/Au core-shell nanoparticles after synthesis (**a**) and the particle size distribution (inset): HAADF micrographs (**b**, **c**) with the corresponding EDX maps of Pt and Au. The arrows indicate the characteristic twinning observed also for pristine gold nanoparticles. The line scan (white dotted line) and elemental distribution is displayed in **d**. **e**) Cyclic voltammetry of gold nanoparticles (upper part) and Pt/Au core-shell nanoparticles (lower part) in 0.1 M HClO_4_ scanning the potential between 0.05 and 1.72 V vs. RHE with a scan rate of 50 mV s^−1^. After the deposition on glassy carbon, carbon monoxide was removed by hydrogen peroxide. The cyclic voltammogram of the as synthesized core-shell particles can be found in Fig. S7.

The concept of colloidal self-limiting growth is displayed in Fig. 1. On the one hand, a metal-ligand system is needed where the binding energies between the metal surface to an appropriate surfactant can provide sufficient colloidal stability in solution while simultaneously allowing further overgrowth of a second metal. Gold as core metal and citric acid (Au/CA) with low core-binding carboxyl-functional groups were chosen. On the other hand, a second metal-ligand system is necessary where the binding energies between the overgrown metal to the surfactant are high while low interactions between the second surfactant and the core are wished for. Here, platinum and carbon monoxide were selected as the second complementary metal-ligand system (Pt/CO). The strong interaction between Pt and CO is known from heterogeneous catalysis where CO acts as catalyst poison. Pt and Au demonstrate similar lattice parameters with gold being slightly larger in size. The surface energies of Pt [fcc (111): 2.35 m^−2^] are slightly larger compared to Au [fcc (111): 1.61 Jm^−2^] and can be altered depending on the respective (ligand) environment^33^. First, spherical Au-nanoparticles with a diameter of (24.9 ± 3) nm were synthesized by means of colloidal chemistry methods^34^ and characterized by (scanning) transmission electron microscopy and cyclic voltammetry shown in Fig. S1 and Fig. 2.

Subsequently, the red-colored citrate capped aqueous gold nanoparticle solution was saturated at room temperature with CO and ascorbic acid (AA) as reducing agent was added and dissolved. After CO-saturation, Pt^IV^ was added that led with time to an intensified red color (c.f. UV-Vis, Fig. S2). Below pH 6, the reduction to Pt^0^ is expected to be pH independent. A Pourbaix diagram from the specific ∆G^0^ for the involved species in aqueous solution is displayed in Fig. S3. The oxidation of ascorbic acid to dehydroascorbic acid (DHA), however, depends strongly on the pH and a pH range between 3 and 4 was found to result in Pt-reduction (Fig. S4). This is in line with Rueda and coworkers who reported that at pH 2 to 4.5, the catalysis of ascorbic acid to dehydroascorbic acid on gold undergoes a two-proton interchange with low overpotentials and sufficiently high reaction rates^35^. The catalysis of both, the reduction of Pt^IV^ and the oxidation of AA has to take place with sufficient reaction rates on the respective metal surface to ensure platinum deposition. After the synthesis of Au-NPs, the surface is covered by citric acid acting as surfactant to stabilize the nanoparticles in solution. The corresponding carboxyl (from citric acid) - gold binding energy was reported to lie in the range of ~0.09 eV and is considered relatively weak^36^. The adsorption and desorption of citrate anions is a dynamic process that has been suggested to even compete with water molecules on Au(111)^37,38^. The low binding energies result in a low citrate surface coverage on gold nanoparticles that goes along with the formation of net-like structures of the citrate layer^36^. The addition of AA lowers the mixed potential established between the Pt^IV^/Pt^II^/Pt^0^- and the AA/DHA redox system. This was monitored in a three-electrode setup by monitoring the open circuit potential (OCP) using a Au-film as working electrode, a graphite rod as counter electrode and a Ag/AgCl reference electrode in 0.1 M HClO_4_. The chronopotentiometric measurements are shown in Fig. S5. Driven by the spontaneous electrochemical reduction of Pt^IV^ and oxidation of AA, platinum deposits on the gold surface and further growth is kinetically hindered by the subsequent adsorption of carbon monoxide. Binding energies of CO to Pt reach up to ~2 eV while CO binding energies to Au are significantly lower (~0.6 eV)^39–42^. The reason lies in the stabilizing charge transfer from platinum to the antibonding 2π* CO molecular orbitals^43^. The synthesis was stopped after 100 min, and the particles were centrifuged and analyzed by high-angle annular dark-field (HAADF) imaging. The HAADF and EDX mapping reveal Pt/Au core-shell nanoparticles with a Pt thickness of 0.76 ± 0.22 nm, corresponding to three to four monolayers as shown in Fig. 2d. The lattice fringes with measured d-spacing of 0.235 nm coincide well with (111) planes of face-centered Au in the core (Fig. S6).

The ratio of Au/Pt was determined with inductively coupled mass spectrometry. Assuming spherical particles, a shell thickness of ~0.79 nm was obtained, fitting well to the EDX elemental distribution of individual particles. The cross-sectional line scan and the elemental distribution can be found in Fig. 2d. After establishing the feasibility of the described synthesis method, the surface was probed via electrochemical methods to obtain first evidence of homogeneous shell formation. Detailed analysis of the cyclic voltammograms between 0.05 and 1.72 V_RHE_ revealed characteristic platinum redox features, including the region of hydrogen underpotential deposition (H_UPD_) between 0.05 < *E* < 0.4 V_RHE_ (R-1, O-1), the adsorption of oxygenated species above 0.65 V_RHE_ or the surface oxidation of Pt above 1.05 V_RHE_ (O-2) and the oxidation of water above 1.6 V_RHE_ (O-3). R-2 corresponds to the reduction of platinum oxide (Pt-O) to Pt^0^ while R-3 corresponds to the gold oxide (Au-O) reduction to Au^0^. During consecutive cycling, the charge associated with the R-2 peak decreases while the peak current and the associated charge of R-3 increases. Analogous, the H_UPD_ associated charge (O-1, R-1) decreases while the gold oxidation (O-3) charge increases. From the voltammograms, the consecutive cycling leads to Pt/Au intermixing that is driven by the decrease in surface energy that accompanies Au surface segregation. The lack of a Au reduction peak in the first cycles hints to a complete Pt coverage and missing electrolyte accessibility to Au. These observations were corroborated by in-situ, time and potential-dependent dissolution studies that were performed via a flow cell (FC) coupled to an inductively coupled mass spectrometer (ICP-MS) as shown in Fig. S8. For pristine Au nanoparticles, the characteristic transient dissolution phenomena were observed when Au is oxidized and reduced during dynamic potential operation^44^. For Pt/Au core-shell nanoparticles, the detection limit of the ICP-MS is reached and only Pt-specific dissolution was observed which is in line with the missing charge associated to R-3 in Fig. 2e. Our results demonstrate that a homogeneous shell growth is possible by the proposed self-limiting growth through CO.

**Fig. 3:**
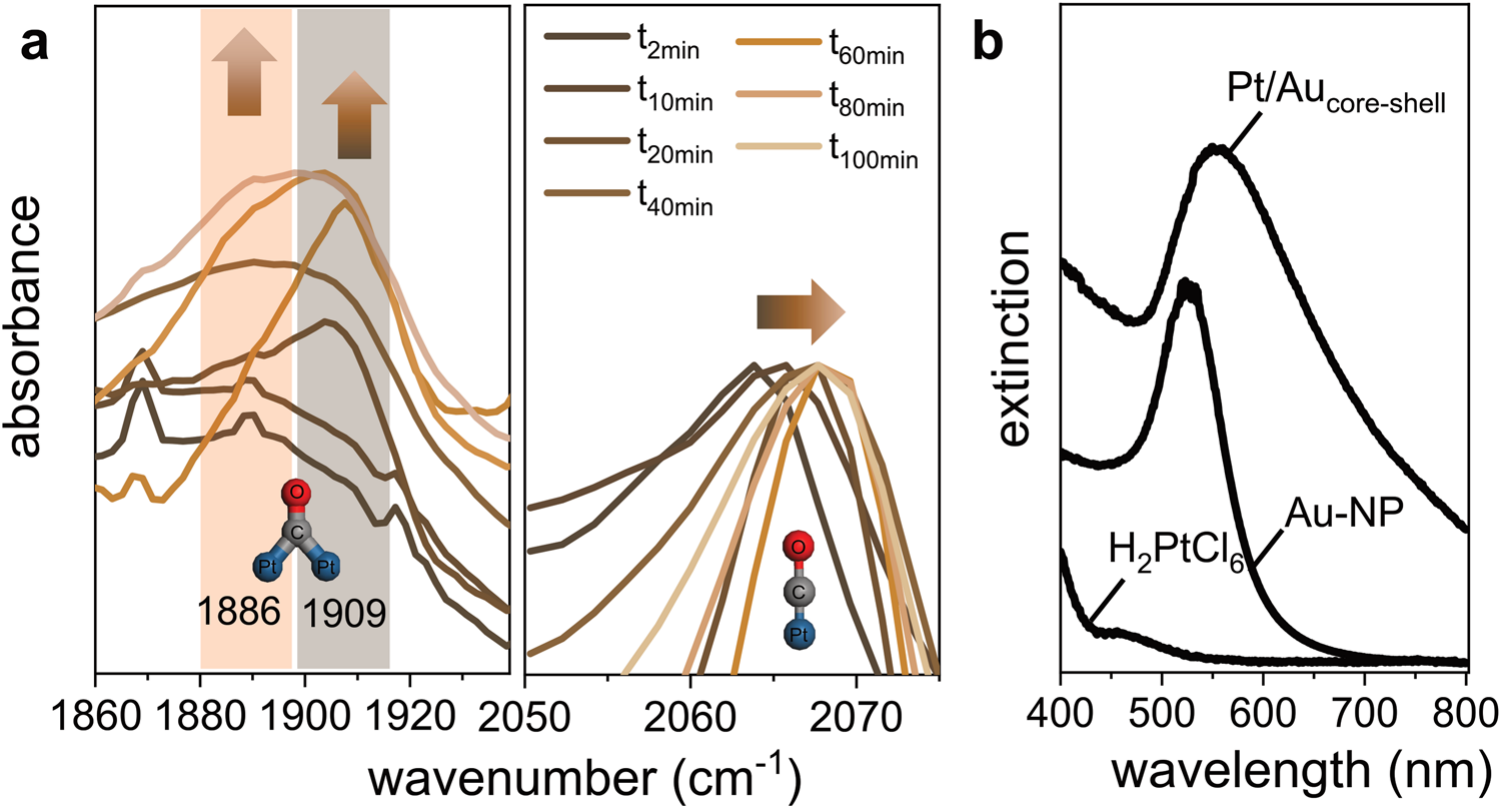
Growth of core-shell Pt/Au nanoparticles. **a** ATR-FTIR analysis of aliquots after different time intervals while the reduction of Pt was quenched in the presence of dissolved CO. **b** UV-Vis spectra of 5 mM H_2_PtCl_6_, citrate-stabilized Au-nanoparticles and Pt/Au core-shell nanoparticles directly after synthesis.

The growth of the layered core-shell structure was further monitored ex-situ by means of attenuated total reflection Fourier transform infrared spectroscopy (ATR-FTIR) as shown in Fig. 3. The high sensitivity of the CO stretching vibration allows for identifying the details of bonding of the molecule to the substrate^43^. The addition of Pt^IV^ was used as reference point (t_0min_). The ATR-FTIR spectrum of citrate capped Au-nanoparticles in the presence of AA and CO reveals broad vibrational peaks at ≈2140 cm^−1^ (CO stretching mode^45^) indicating weak interactions of CO with Au (Fig. S9). After addition of Pt^IV^, while saturating the solution further with CO, several time-dependent ATR-FTIR spectra were recorded. After t_2min_, the solution exhibits a strong vibrational band at 2062 cm^−1^ attributed to terminal carbonyls bound in an a-top geometry on Pt^46–48^. With time, an additional band at 1909 cm^−1^ appears with a growing shoulder at 1886 cm^−1^, both attributed to bridged CO in Pt–CO–Pt species^49^. We assigned the band at lower wavenumbers to the formation of Pt-carbonyl, that were also observed when no Au-nanoparticles were present. A detailed discussion of the simultaneously formed Pt-carbonyl complex and the associated UV-Vis and IR-bands can be found in Figs. S2, S10–S12 and in the Supplementary Methods. The formation of a peak at higher wavenumbers indicates continuing Pt deposition. When the gradual deposition of Pt on Au continues, the Pt-layer smoothens. The decreased Pt d-electron back-donation to the CO π antibonding orbitals results in the accompanied blue shift from 2062 cm^−1^ to 2068 cm^−1^ ^50^. The observed blue shift stems from changes in dipole-dipole coupling between CO molecules in the surface overlayer. Blue-shifted peaks of cationic Pt^δ+^- or single atom Pt-carbonyl, expected in the region between 2090 to 2150 cm^−1^, were not observed^51^. After passivation of the Pt-surface by CO, the growth of Pt on Pt slows down and Pt heterogeneously nucleates and wets further electrolyte facing Au-facets. From DFT calculations on single-crystal surfaces, the binding energy of CO to Pt on Au(111) surfaces reaches almost 2 eV directly after the first Pt-monolayer is formed and does not increase exponentially with thickness^52,53^. Despite the large Pt-CO binding energies, CO-binding is a dynamic process where surface diffusion, desorption and re-adsorption on the Pt-surface can take place. CO was reported to be mobile above 100 K and to exhibit a saturation coverage on Pt(111) surfaces of Θ = 0.6 in the aqueous environment and under ultra-high vacuum^54,55^. ATR-FTIR spectroscopy indicates that full Pt-coverage is reached after ≈20 min as a further peak shift was not observed.

Strong indication of our hypothesis of a CO concentration-dependent deposition was gained when ethanol instead of water was used as solvent. Here, the CO-solubility increases by a factor of seven compared to aqueous solution and the shell thickness decreases to ~0.75 monolayers (ML) after t_100min_ of Pt-deposition (Fig. S13)^56^. We note that the degree of solvation of both, the ions and the surface is highly dependent on the type of solvent resulting in different surface charges, *ζ* potentials and varied deposition characteristics independent of the amount of dissolved CO influencing further the deposition. Analogous to monometallic colloidal particles, the described synthesis route yields stable and “unprotected” nanoparticles that can be used as tractable building blocks in e.g. catalysis. The term “unprotected” was used to emphasize the stabilization only by solvent molecules or easily oxidizable ligands such as CO^57–60^.

**Fig. 4:**
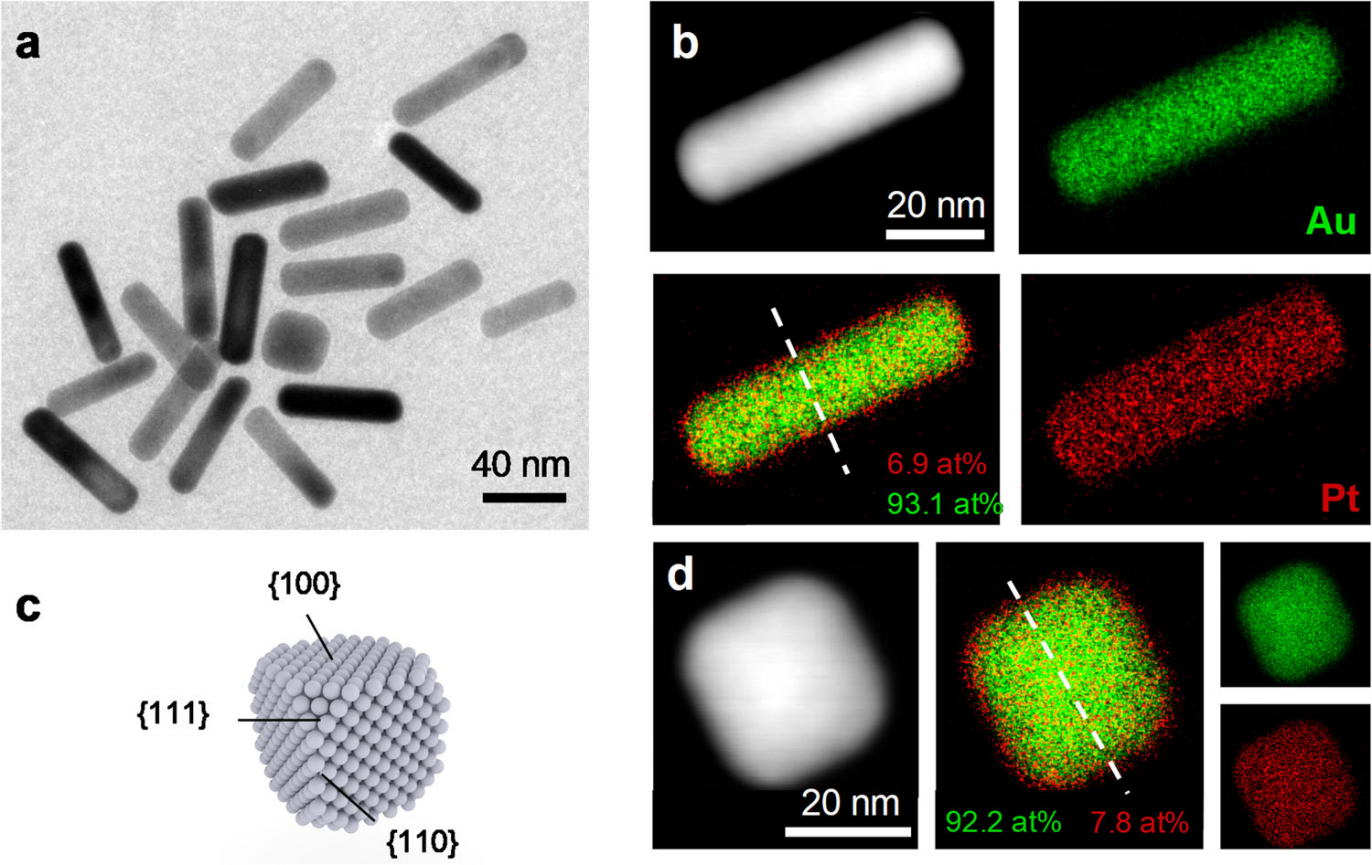
Self-limited growth of a Pt-shell on shape-controlled nanoparticles. **a** TEM images of nanorods and nanocubes covered by a, in the subnanometer range lying Platinum shell. **b**, **d** HAADF images of a singular nanorod and nanocube and the respective Pt (red) and Au (green) elemental distribution. The respective crystal facets for cubic nanoparticles is displayed in **c**. The elemental line-scans can be found in Fig. S14 (white dotted lines).

After establishing the growth of spherical nanoparticles covered by Pt, the concept was expanded to nanoparticles with defined facets. In particular, Au nanorods (NR) and nanocubes (NC) capped with citrate ligands were used for the subsequent overgrowth as shown in Fig. 4. The Au{111} surface is typically the most populated facet for large spherical Au nanoparticles^61^ followed by the {100} surface^36^, which is in line with the observed icosahedral shape of the synthesized nanoparticles (Fig. S6), while the Au{100} dominates in cubic- (Fig. S15) and Au{110}/Au{100} surfaces dominates in rod-shaped nanoparticles^62,63^. Using similar synthesis conditions as for spherical nanoparticles (c.f. Supplementary Methods), the growth of a Pt-overlayer was observed for all materials (Figs. S15–S17). A preferential influence in Pt-thickness due to the longitudinal growth direction parallel to the Au{001} plane and the higher amount of Au{100} and Au{110} surfaces could not be observed by STEM-EDX. The same holds for the cubic nanoparticles with a high number of Au{100} crystal facets. The difference in facet specific surface energies of Au [e.g. Δφ(100)−(111)] lies with over 0.1 eV higher than Au-carboxylate interaction^36,64,65^, however is an order of magnitude lower than the dominating Pt-CO interaction. The made observations could potentially apply to other metals such as Pd, Ir or Ru exhibiting strong CO-metal interactions and a wide range of non-noble core materials. Potentially, the formed core-shell particles can be used in fuel cell applications where stabilization and lower Pt dissolution was observed when combined with Au^11,66^.

## Conclusion

In conclusion, we propose a concept to synthesize core-shell nanostructures. The concept relies on the interaction of a nanoparticle surface with labile ligands followed by the deposition of an outer metal layer and the subsequent self-limiting nature of strong-binding ligands to stop further growth. We applied the synthesis strategy exemplarily to Pt that was deposited on various Au-nanostructures such as spherical nanoparticles, nanocubes and nanorods with differently exposed crystal facets. The self-limited overgrowth observed for spherical and shape-controlled nanoparticles offers a way of engineering the nanostructure of nanoparticles for a wide range of potential applications. By in-situ FC-ICP-MS testing as well as ex-situ STEM-EDX and ATR-FTIR measurements, the Pt-layer was found to be homogenously distributed over the Au-surface. No preferential growth on Au{111}, Au{100} or Au{110} surfaces was observed. With the complementary use of carbon monoxide and ascorbic acid – both accessible on the commodity scale – a cost-efficient strategy to potentially scale up the described methodology is proposed to obtain homogenous and smooth Pt/Au core-shell structures. We envision that the described synthesis concept of stable and “unprotected” core-shell nanoparticles leads to other core-shell nanostructures by selecting a proper self-limiting agent that is tailored to the specific requirements of the core and shell material.

## Methods

### Materials

Hydrogen hexachloroplatinate(IV) hydrate (H_2_PtCl_6_·H_2_O, 99.9%), tetrachloroauric acid (HAuCl_4_·3H_2_O, 99.9%), ascorbic acid (AA), and trisodium citrate dihydrate were purchased from (Sigma)-Aldrich and used as received. Milli-Q water (18.2 MΩ.cm) was used in all synthesis procedures. All materials were used without further purification unless specified otherwise in the experimental methods.

### Material preparation

#### Spherical Gold nanoparticles

The synthesis of Au-NP was adapted from Turkevich et al.^34^ Prior to use, all glassware was cleaned with aqua regia and rinsed with Milli-Q water. First, 0.026 g HAuCl_4_*3H_2_O was dissolved in 50 ml ultrapure water in an Erlenmeyer flask and heated on a stirring hot plate until boiling. Afterwards, a stock solution of trisodium citrate dihydrate (0.5 g) in 50 ml water was prepared. To the rapidly stirred boiling gold solution, 4.5 ml of the prepared stock solution was rapidly added and heat supply was provided for additional 10 min. The yellow solution turned deep red and were cooled down to room temperature. The obtained particles are displayed in Fig. S1.

### Spherical gold core, platinum shell nanoparticles

Ascorbic acid (Sigma Aldrich, 75 mg) was used in excess and was dissolved in the red-colored citrate capped Au nanoparticles solution (2 ml) as reducing agent and the solution was saturated with CO for 10 min. Afterwards, 2 ml of H_2_PtCl_6_ (5 mM) was added. After 10 min, an intensified red color could be observed (c.f. Scheme S1 and Fig. S15). We note that CO can act as reducing agent for noble metals itself. Without the addition of AA, no color change was observed when the solution was saturated with CO for 2 h (Fig. S18), indicating that the reduction of Pt^IV^ to Pd^0^ by CO is kinetically hindered at the here employed conditions. The addition of ascorbic acid, with lower reduction potential, was found to accelerate the noble metal deposition onto the gold core. Presumably due to the changed pH after addition of AA, the reduction to Pt^0^ is accelerated as expected as shown in Fig. S3. At the same time, the oxidation of L-ascorbic acid on gold has been shown to be first order with respect to L-ascorbic acid and changes linearly with pH at low potentials (59 mV per pH)^35^. The monolayer thickness (ML) was determined by translating the Pt-thickness obtained from STEM/ESX line scans by dividing it by the Pt (1 1 1) plane distance of 0.22 nm.^[13]^ The high binding energies of CO to the Pt-surfaces hinders further growth even when platinum is used in excess as the solubility of CO in water is rather poor with 27.6 mg/L (25 °C, H_2_O). The solution was saturated further with CO for t_100min_. Afterwards, the solution was centrifuged. The centrifugation speed was altered starting from 3000 rpm for 5 min and increased every time 1000 rpm further up to 10 000 rpm. The larger Pt/Au nanoparticles sediment first while the Pt-carbonyl complex stays in solution. When the solution is not centrifuged and stored under ambient conditions, the Pt-carbonyl complex decomposes after a few hours and small Pt-nanoparticles in the size range of 1–2 nm were observed as shown in Fig. S9. This is in line with experiments without gold nanoparticles present where the H_2_PtCl_6_ solution was saturated first with CO and afterwards left overnight in air (Fig. S19). Clearly, the formation of platinum nanoparticles can be observed when no CO is present. We tried to accelerate the sedimentation by adding the same solution amount of THF as antisolvent. The particles immediately sedimented. However, a redispersion afterwards was not possible. By tuning the THF/H_2_O ratio, an accelerated sedimentation seems likely. When sodium borohydride was used as reducing agent (instead of AA), the homogeneous reduction of Pt^IV^ to Pt^0^ was promoted and the solution turned dark after several seconds. In case of sodium borohydride, the binding of CO to Pt seems not sufficient to prevent homonucleation of single Pt-particles. While the deposition of Pt^IV^ to Pt^0^ is not expected to depend on the pH, ascorbic acid (AA) on gold oxidizes at lower potentials when increasing the pH (59 mV per pH) as shown in Fig. S3. The pH dependent formation of pristine Pt-nanoparticles without CO as self-limiting agent is shown in Fig. S20. Using the described synthesis conditions, the solution was found to contain H^+^ concentrations of 10^−4 ^mol/l.

### Shape-controlled core-shell nanoparticles

Citrate capped nanorods and nanocubes were obtained from nanopartz. The nanorods had a diameter of 10 nm, length of 41 nm, optical density (OD) of 1 and aspect ratio of 4.1. The wt. concentration was 35 µg/ml. The solution was further concentrated to 150 µg/ml. The Pt-overgrowth was performed analogous to its spherical counterpart. In short, 1 ml of NR solution was saturated with CO and 37.5 mg of ascorbic acid was dissolved. After 10 min of CO saturation, 1 ml of the platinum solution was added and left for 100 min saturating the solution further with CO. At around 30 min, the nanorods/nanocubes sedimented to the ground.

A detailed description of the characterization methods employed can be found in the SI.

## Supplementary information

Supplementary Information

## Data Availability

The data that support the findings of this study are available from the corresponding authors upon reasonable request.
