# Supplementary material for: Engineering gold-platinum core-shell nanoparticles by self-limitation in solution
Source: Commun Chem. 2022 Jun 6;5:71. doi: 10.1038/s42004-022-00680-w (PMC9814372; doi:10.1038/s42004-022-00680-w)
Supplement: Supplementary file 1 — Supplementary Information [file 42004_2022_680_MOESM1_ESM.pdf]

Supporting information

Engineering gold-platinum core-shell nanoparticles by self-limitation in solution

Marc Ledendecker[a*], Paul Paciok[b], Wojciech T. Osowiecki[c], Marc Pander[d], Marc Heggen[b], Daniel Göhl[a], Gaurav A. Kamat[c], Andreas Erbe[e], Karl J. J. Mayrhofer[f,g], A. Paul Alivisatos[c,h,i]

[a]Department of Technical Chemistry I, Technical University Darmstadt, Alarich-Weiss-Straße 8, 64287 Darmstadt, Germany
[b]Ernst Ruska-Centre for Microscopy and Spectroscopy with Electrons and Peter Grünberg Institute, Forschungszentrum Jülich GmbH, 52425 Jülich, Germany
[c]Department of Chemistry, University of California, Berkeley, California 94720, United States
[d]Department of Interface Chemistry and Surface Engineering, Max-Planck-Institut für Eisenforschung GmbH, Max-Planck-Straße 1, 40237 Düsseldorf, Germany
[e]Department of Materials Science and Engineering, NTNU, Norwegian University of Science and Technology, 7491 Trondheim, Norway
[f]Forschungszentrum Jülich, Helmholtz-Institute Erlangen-Nürnberg for Renewable Energy (IEK-11), Egerlandstraße 3, 91058 Erlangen, Germany
[g]Department of Chemical and Biological Engineering, Friedrich-Alexander-Universität Erlangen-Nürnberg, Egerlandstraße 3, 91058 Erlangen, Germany
[h]Department of Materials Science and Engineering, University of California, Berkeley, California 94720, United States
[i]Kavli Energy NanoScience Institute, Berkeley, California 94720, United States
*Corresponding author: marc.ledendecker@tu-darmstadt.de

Synthesis

Au-nanoparticles

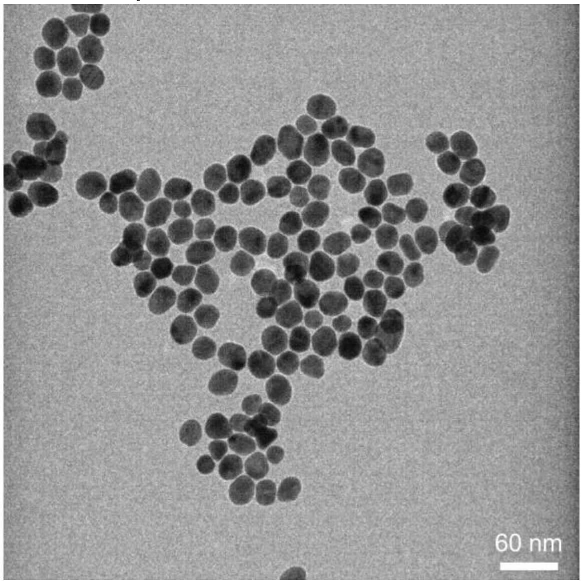

Figure S1: Electron microscopy image of pristine gold-nanoparticles synthesized using sodium citrate as reducing agent.

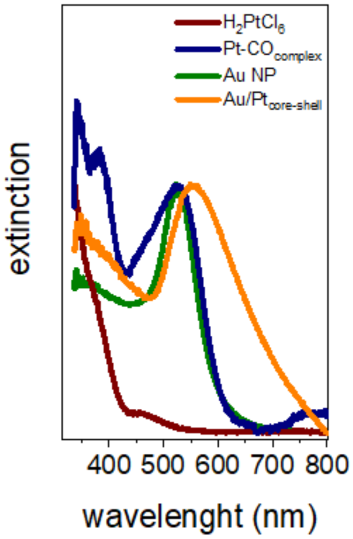

Figure S2: UV-Vis spectrum from 5mM H_2PtCl_6 (wine), the formed Pt-CO complexes without Au nanoparticles present (blue), freshly synthesized citrate capped Au-nanoparticles (green) and Pt on Au core-shell nanoparticles (orange) prior to washing.

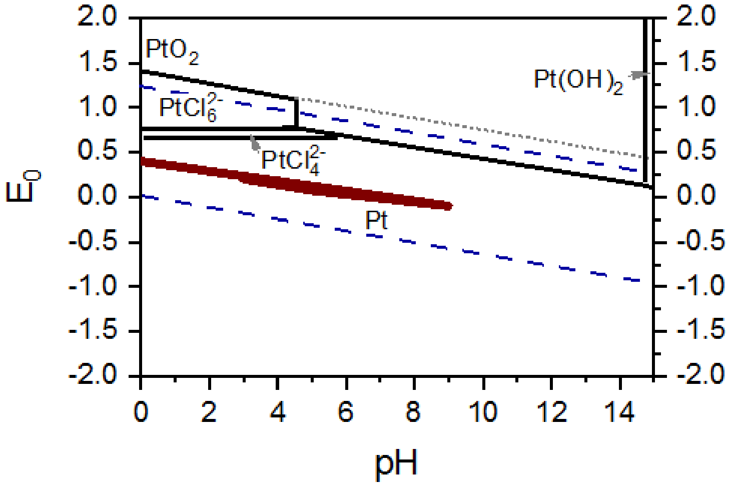

Figure S3: Pourbaix diagram for Pt/Cl in aqueous solution. The diagram was composed from Reference[2] *for the Pt/Cl system. The red line corresponds to the ascorbic acid/ dehydro ascorbic acid system.*[3] *A Pt concentration for soluble species of 10^{-3} M was used. The concentration for Cl soluble species were 1M. The used specific ΔG^0 can be found in* [2]*.*

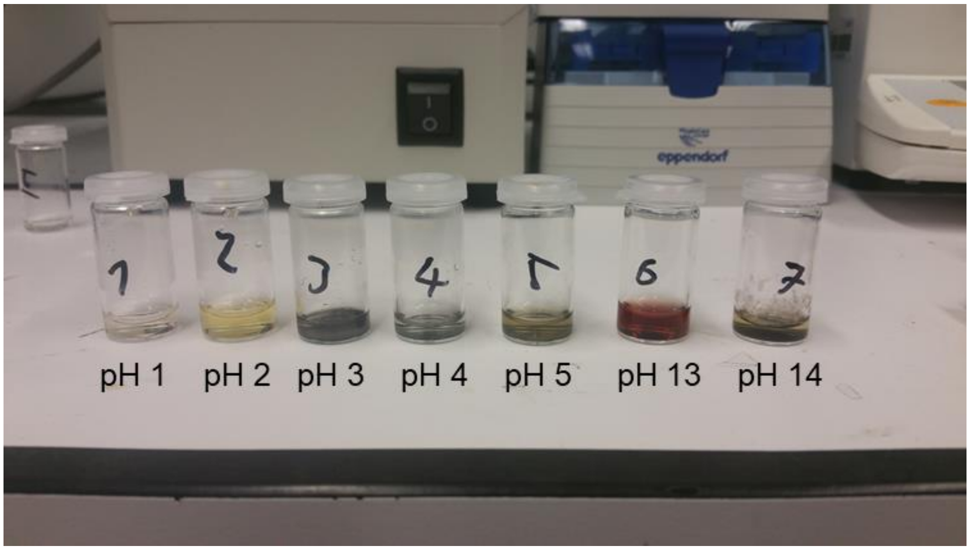

Figure S4: Aqueous H_2PtCl_6 (5 mM) under different pH conditions (adjusted with HCl or NaOH, suprapure) after the addition of AA (75mg) and a reaction time of 100 min.

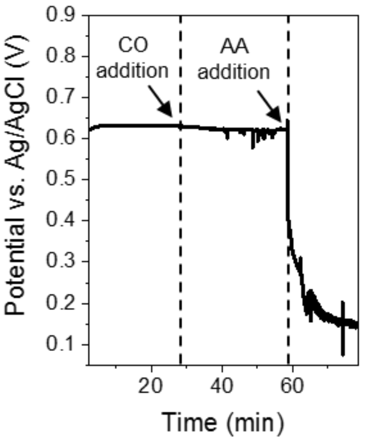

Figure S5: Bare gold substrate immersed in aerated 0.1M $HClO_4$ in a three-electrode setup where the open circuit potential was monitored. A Ag/AgCl$_{sat.}$ reference electrode and a graphite rod as counter electrode were employed. The electrochemical cell was an in-house made Teflon cell. A Nafion membrane separated the working and counter electrode compartment. The change in OCP was monitored after the addition of CO and AA as indicated by the dashed lines.

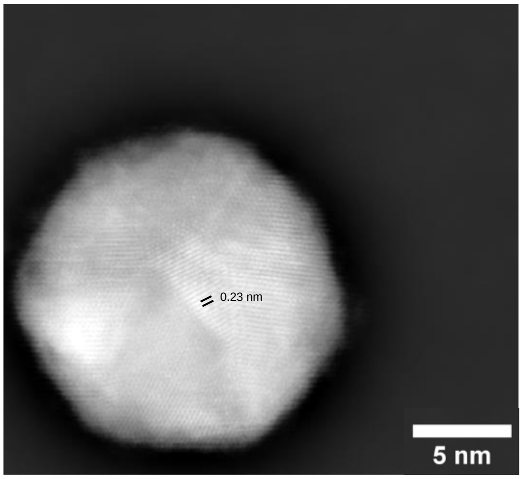

Figure S6: Transmission electron micrograph of a single Au nanoparticle after the overgrowth of Pt. The atomic lattice fringes of Au are clearly resolved.

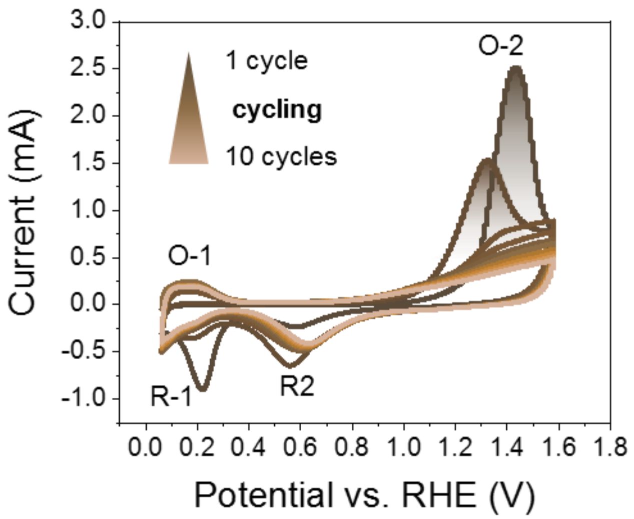

Figure S7: Potential cycling of Au/Pt core-shell nanoparticles directly after synthesis between 0.05 and 1.58 V_{RHE} in 0.1M $HClO_4$ with a scan rate of 200 mV s^{-1}. The first scans reveal the characteristic peaks in the CO-stripping region and missing peaks in the H_{UPD} region.

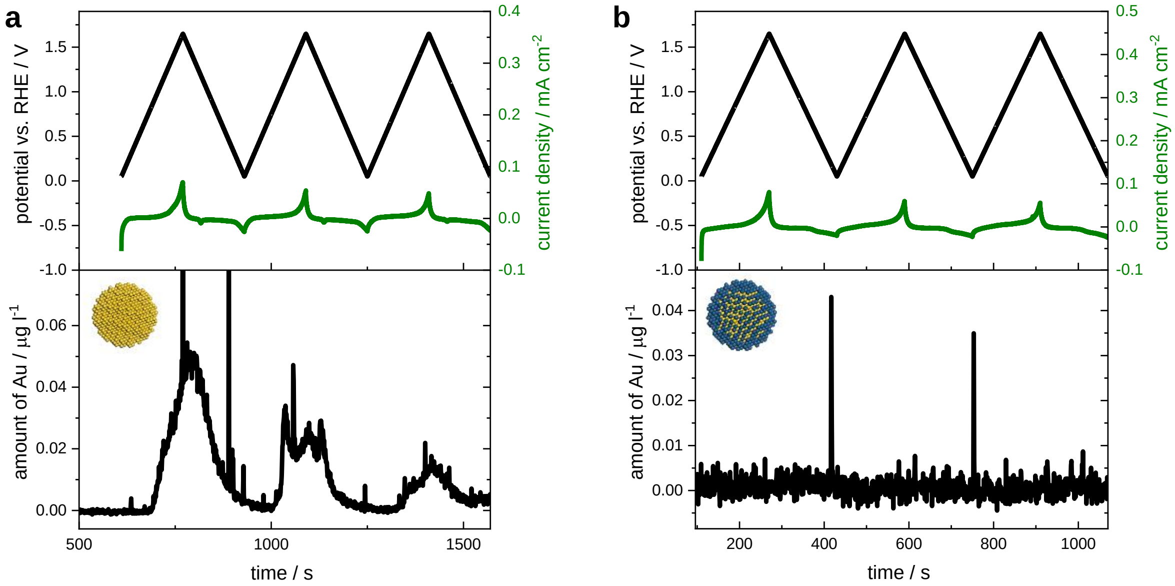

Figure S8: On-line FC-ICP-MS measurements with pristine Au-nanoparticles (a) and core-shell Au/Pt nanoparticles (b) while scanning the potential from 0.05 to 1.65 V_{RHE} with a scan rate of 10 mV s^{-1} in 0.1M $HClO_4$.The potential-current diagrams are displayed on the top while the potential dependent dissolution of gold is displayed at the bottom. Spikes in the dissolution diagram correspondents to single particles that detached during measurements.[10]

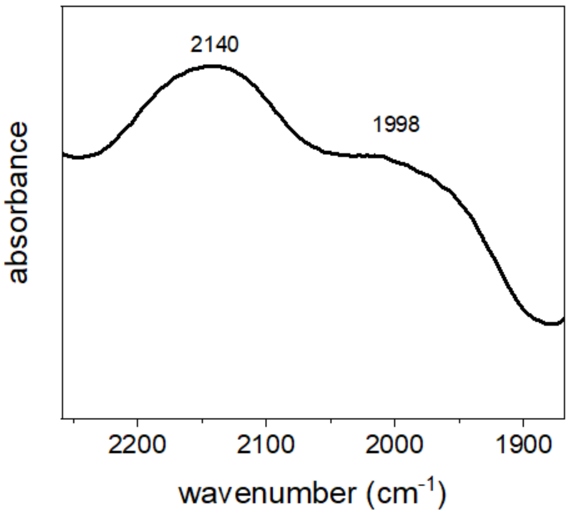

Figure S9: ATR-FTIR spectrum of Au-nanoparticles in the presence of CO and AA in aqueous medium. The Au-nanoparticles and AA background (Ar-purged) was subtracted.

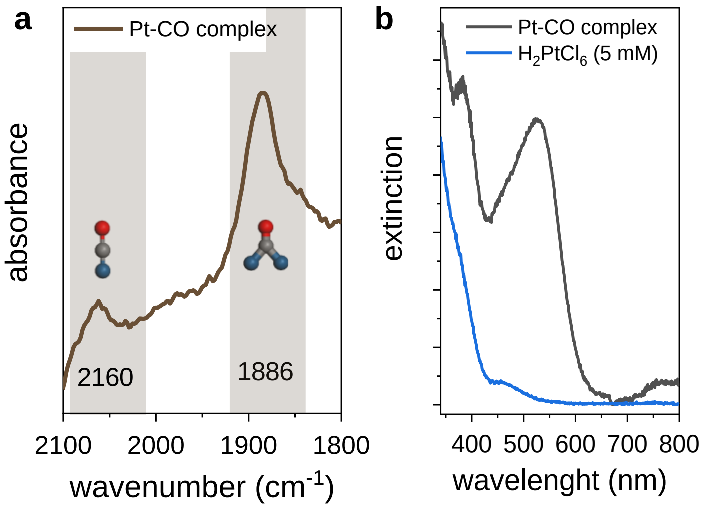

Figure S10: a) ATR-FTIR spectrum of the CO saturated H_2PtCl_6 solution after addition of AA and a reaction time of 30 min (b). UV-Vis spectrum of the formed Pt-CO complex and the comparison to H_2PtCl_6 in aqueous solution.

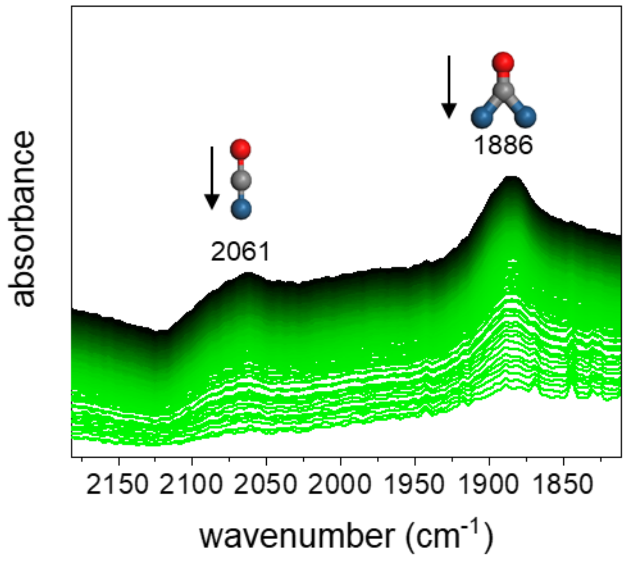

Figure S11: ATR-FT-IR spectra of the formed Pt-$CO_{complex}$ (after 30 min) through the addition of AA to a CO saturated solution of H_2PtCl_6 solution. Different spectra were recorded directly after synthesis (black) and after stopping CO saturation (green) in a time frame of 1h.

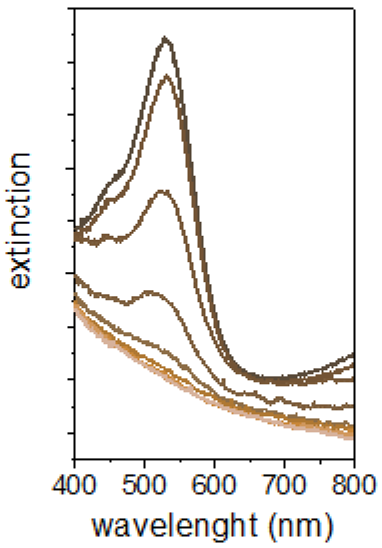

Figure S12: Monitoring the decomposition with time of the formed Pt-CO complexes by H_2O_2 using UV-Vis spectroscopy. Beginning of life (black) and end of life (light brown). Each spectrum was taken with a two minutes' time delay to the previous spectrum.

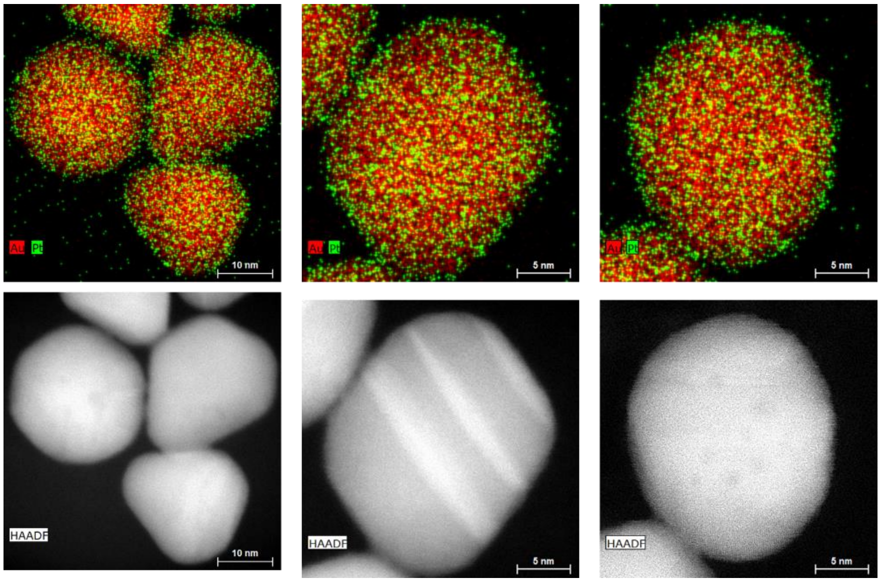

Figure S13: HAADF images of core-shell particles synthesized in ethanol with higher CO-solubility compared to H_2O and the respective EDX mapping results (Pt: green, Au: red).

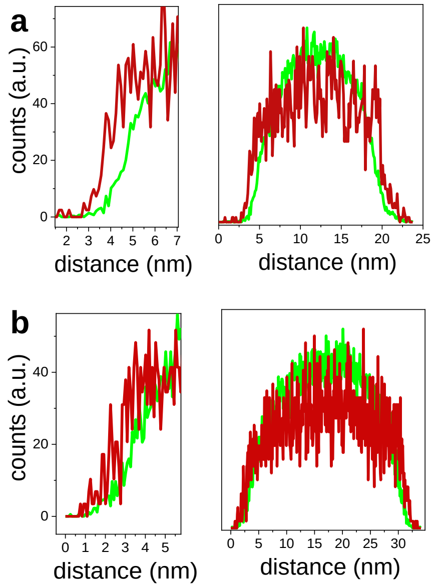

Figure S14: EDX-line scans of (a) Au/Pt nanorods and b) Au/Pt nanocubes from Figure 3.

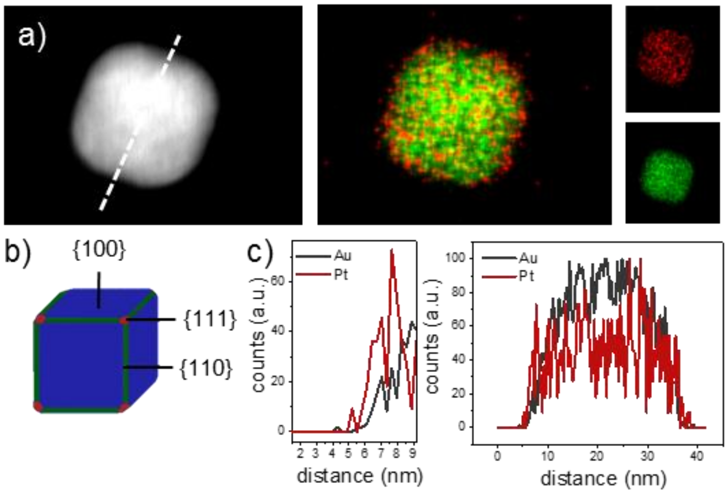

Figure S15: HAADF-EDX images of a single Pt/Au nanocube (a), the respective Au (green) and Pt (red) element distribution, the expected cubic particle with the corresponding facets (b) and the line scan and element count according to the white, dotted line in a).

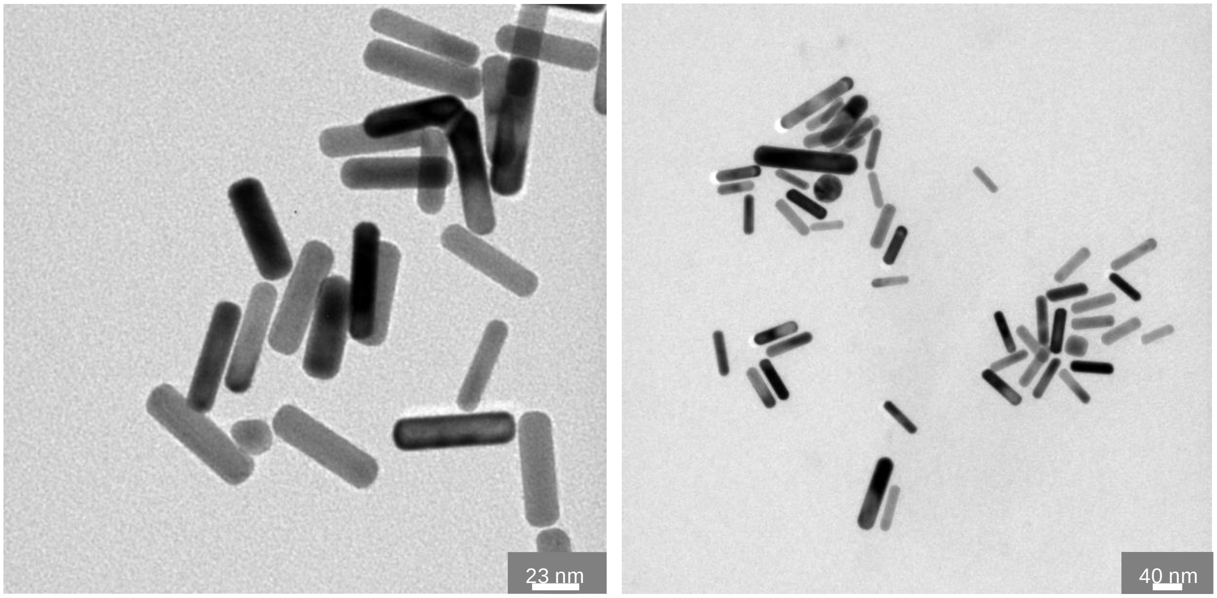

Figure S16: TEM images of Au-nanorods and occasionally occurring Au-nanocubes after the growth of a thin Pt-overlayer.

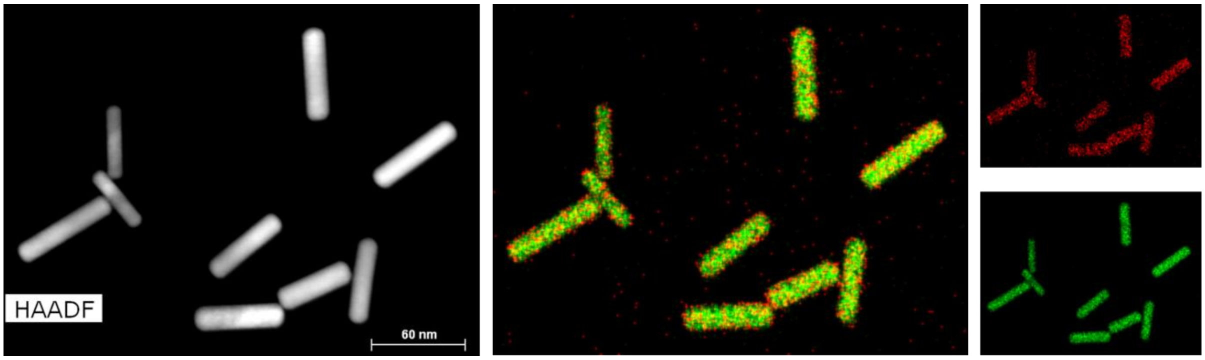

Figure S17: HAADF-EDX images of Au-nanorods (green) and a thin layer of overgrown Pt (red)

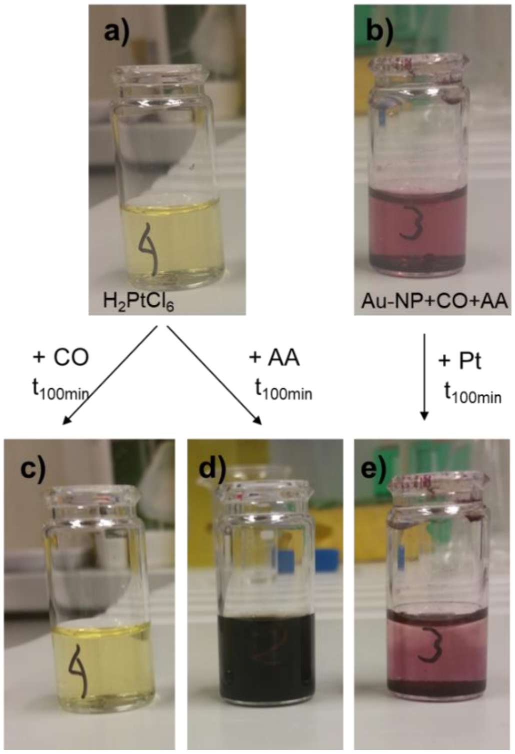

Figure S18: H_2PtCl_6 (5mM) before (a) and after the addition of CO for 100 min (c) and in the presence of 75 mg AA (d, no CO saturation). Au-nanoparticles (2 ml) and 5 mM H_2PtCl_6 before (b) and after the addition of AA while saturating the solution further with CO (e). The reaction time for all samples was set to 100 min.

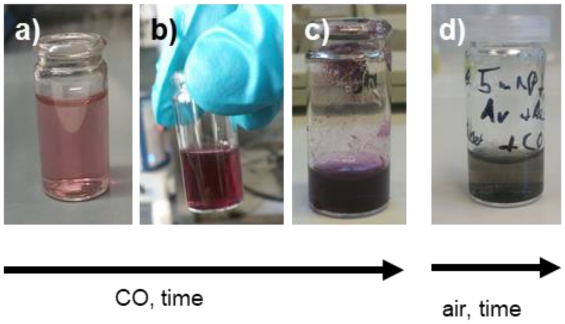

Figure S19: CO saturated solution of H_2PtCl_6 after the addition of AA after a) 10 min, b) 30 min and c) 2h (no gold). The same solution (c) left overnight in air leads to platinum nanoparticles as indicated by the darkened solution (d).

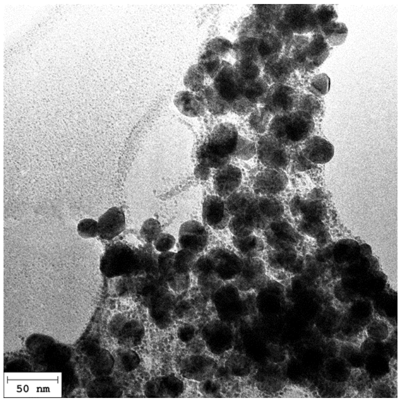

Figure S20: TEM images of Au/Pt core-shell particles and smaller Pt nanoparticles/clusters with no work-up and leaving the prepared Au/Pt solution under ambient conditions over night. The smaller nanoparticles next to the larger Au/Pt core-shell nanoparticles were attributed to bare platinum nanoparticles.

Supplementary Methods

Pt-carbonly complex

In order to distinguish between the formed Pt-carbonyl complex and Pt/Au core-shell nanoparticles, the Pt-carbonyl complex was investigated separately and without Au-nanoparticles present. Therefore, 2mL H_2PtCl_6 (5 mM) was saturated with CO for 10 min. After addition of AA (75 mg), the solution turned red and distinct IR bands at 1886 cm^{-1} and 2060 cm^{-1} were observed attributed to bridged and linearly bound Pt-CO species (Figure S10). The strengthened absorption of the bridging CO groups (v_{br} = 1860 cm^{-1}) and weakened terminal CO bands (v_t = 2060 cm^{-1}) might indicate the formation of $[Pt_{3n}(CO)_{6n}]^{2-}$ clusters with n=2-6.[1-3] These cluster have distinct π-π* transition bands depending on n. From UV-Vis spectra, the formation of $[Pt_{3n}(CO)_{6n}]^{2-}$ clusters with n = 2-3 is likely with transitions at 468 nm, 524 nm and 382 nm corresponding well to reported literature values.[4] Also the formation of Pt nanoparticles in the size range of 1-2 nm was observed when CO was present and may also contribute to the spectrum.[5] To shed more light on the nature of the prepared species, the decomposition of the prepared cluster over time by H_2O_2 results in the initial yellow colored H_2PtCl_6 solution as shown in Figure S12.

When the cluster synthesis was performed in ethanol [10 ml EtOH, CO sat., 75 mg AA, 2 ml H_2PtCl_6 (5 mM)], a change in color was not observed indicating that the Pt-CO complex did not form.

Characterisation

Transmission electron microscopy

A Tecnai G2 T20 S-TWIN with a Gatan SC200 CCD camera was used for transmission electron microscopy (TEM). The particle containing solution was drop-casted on a TEM grid (Electron Microscopy Sciences, CF-400-Cu) and images were acquired at 200 kV. For high-resolution scanning TEM (HR-STEM) imaging, either a JEOL 2100-F or a FEI TitanX were used, both operating at 200 kV. STEM-HAADF and STEM-EDS imaging were performed on FEI TitanX using a Fischione high-angle annular dark-field (HAADF) detector. A FEI Super-X windowless detector was used as EDS detector. For quantitative elemental analysis, Bruker Esprit was used. Ultrathin carbon on gold TEM grids (Ted Pella, 01824G) were cleaned (oxygen-plasma) prior to particle drop-casting for high resolution ensuring low carbon contamination. A Shimadzu UV-3600 UV–Vis–NIR spectrophotometer was used for UV-Vis spectroscopy of the colloidal suspension in water in quartz cuvettes.

Spectroscopic electrochemical setup

To conduct spectroscopic measurements, the solutions were placed in the beam path of a FT-IR Spectrometer (FTS 3000 MX Excalibur Series, Bio-Rad Laboratories, Inc.) in an ATR-IR setup.

Cyclic voltammetry

A three-compartment electrochemical Teflon cell was used. A saturated $Ag/AgCl_{sat}$ electrode (Metrohm, Filderstadt, Germany) served as reference electrode while a graphite rod served as counter electrode. As electrolyte, 0.1M $HClO_4$ was used and the potential was converted to the reversible hydrogen electrode scale. The potential was measured before every measurement by saturating the corresponding electrolyte solution with hydrogen gas using a platinum working electrode.

The voltammogram of CO-capped Au/Pt core-shell nanoparticles directly after synthesis in 0.1M $HClO_4$ is shown in Figure S18. During the first cycles, no peaks associated to hydrogen adsorption/desorption were observed while an oxidation charge at potentials between 1.2 and 1.5 V_{RHE} were observed. CO-stripping usually takes place at potentials below 1V_{RHE}. It is speculated

that the oxidation charge stems from oxidation of CO as well as from other organic molecules. On the reductive back cycling, one sharp reduction peak at around 0.2 V_{RHE} was observed, presumably from the reduction of organic molecules still present. After the first cycle, the charge associated to underpotential deposited hydrogen increases while the charge associated with CO oxidation decreases. After two cycles, the characteristic Pt-oxidation and Pt-O reduction peaks appear (c.f. main text).

Scanning flow cell coupled to an ICP-MS

The scanning flow cell was coupled with an inductively coupled plasma mass spectrometer (ICP-MS, NexION 300X, Perkin Elmer) as described elsewhere.[6] A polycarbonate-based scanning flow cell was home-made with a CNC machine. The working electrode opening was 0.01 cm^2. An $Ag/AgCl_{sat}$. (Metrohm, Germany) electrode was used as reference electrode while a graphite was used as counter electrode respectively. The Ag/AgCl reference electrode was calibrated against a reversible hydrogen electrode (platinum foil, $H_{2\text{-}sat}$ 99.99%, MaTeck, Germany) prior to each measurement. As electrolyte, 0.1 M $HClO_4$ was used (Diluted with ultrapure water (PureLab Plus system, Elga, 18 MΩ cm^{-1}, TOC < 3 ppb) from concentrated $HClO_4$, Merck, Suprapure®). As electrolyte, 0.1 M $HClO_4$ was used and purged with Ar. The flow rate from the ICP-MS was 190 µL min^{-1} through the SFC. A daily performance test was conducted on the ICP-MS to ensure proper working conditions. The ICP-MS was calibrated prior to each measurement sequence. A four-point calibration was performed before each measurement sequence.

Supplementary References:

1 Fukuoka, A. *et al.* Ship-in-bottle synthesis and catalytic performances of platinum carbonyl clusters, nanowires, and nanoparticles in micro- and mesoporous materials. *Catalysis Today* **66**, 23-31, doi:https://doi.org/10.1016/S0920-5861(00)00601-5 (2001).

2 Calabrese, J. C., Dahl, L. F., Chini, P., Longoni, G. & Martinengo, S. Synthesis and structural characterization of platinum carbonyl cluster dianions bis,tris,tetrakis, or pentakis(tri-.mu.2-carbonyl-tricarbonyltriplatinum)(2-). New series of inorganic oligomers. *Journal of the American Chemical Society* **96**, 2614-2616, doi:10.1021/ja00815a050 (1974).

3 Longoni, G. & Chini, P. Synthesis and chemical characterization of platinum carbonyl dianions [Pt3(CO)6]n2- (n = .apprx.10,6,5,4,3,2,1). A new series of inorganic oligomers. *Journal of the American Chemical Society* **98**, 7225-7231, doi:10.1021/ja00439a020 (1976).

4 Rabilloud, F., Harb, M., Ndome, H. & Archirel, P. UV−Visible Absorption Spectra of Small Platinum Carbonyl Complexes and Particles: A Density Functional Theory Study. *The Journal of Physical Chemistry A* **114**, 6451-6462, doi:10.1021/jp912117q (2010).

5 Surendran, G. *et al.* From Self-Assembly of Platinum Nanoparticles to Nanostructured Materials. *Small* **1**, 964-967, doi:10.1002/smll.200500011 (2005).

6 Klemm, S. O. *et al.* Time and potential resolved dissolution analysis of rhodium using a microelectrochemical flow cell coupled to an ICP-MS. *Journal of Electroanalytical Chemistry* **677–680**, 50-55, doi:http://dx.doi.org/10.1016/j.jelechem.2012.05.006 (2012).
